# The potential for prevention of dementia across two decades: the prospective, population-based Rotterdam Study

**DOI:** 10.1186/s12916-015-0377-5

**Published:** 2015-07-21

**Authors:** Renée FAG de Bruijn, Michiel J Bos, Marileen LP Portegies, Albert Hofman, Oscar H Franco, Peter J Koudstaal, M Arfan Ikram

**Affiliations:** Department of Epidemiology, Erasmus University Medical Center, Wytemaweg 80, 3015 CN Rotterdam, The Netherlands; Department of Neurology, Erasmus University Medical Center, ’s-Gravendijkwal 230, 3015 CE Rotterdam, The Netherlands; Department of Radiology, Erasmus University Medical Center, ’s-Gravendijkwal 230, 3015 CE Rotterdam, The Netherlands

**Keywords:** Dementia, Epidemiology, Population attributable risk, Risk factors

## Abstract

**Background:**

Cardiovascular factors and low education are important risk factors of dementia. We provide contemporary estimates of the proportion of dementia cases that could be prevented if modifiable risk factors were eliminated, i.e., population attributable risk (PAR). Furthermore, we studied whether the PAR has changed across the last two decades.

**Methods:**

We included 7,003 participants of the original cohort (starting in 1990) and 2,953 participants of the extended cohort (starting in 2000) of the Rotterdam Study. Both cohorts were followed for dementia until ten years after baseline. We calculated the PAR of overweight, hypertension, diabetes mellitus, cholesterol, smoking, and education. Additionally, we assessed the PAR of stroke, coronary heart disease, heart failure, and atrial fibrillation. We calculated the PAR for each risk factor separately and the combined PAR taking into account the interaction of risk factors.

**Results:**

During 57,996 person-years, 624 participants of the original cohort developed dementia, and during 26,177 person-years, 145 participants of the extended cohort developed dementia. The combined PAR in the original cohort was 0.23 (95 % CI, 0.05–0.62). The PAR in the extended cohort was slightly higher at 0.30 (95 % CI, 0.06–0.76). The combined PAR including cardiovascular diseases was 0.25 (95 % CI, 0.07–0.62) in the original cohort and 0.33 (95 % CI, 0.07–0.77) in the extended cohort.

**Conclusions:**

A substantial part of dementia cases could be prevented if modifiable risk factors would be eliminated. Although prevention and treatment options of cardiovascular risk factors and diseases have improved, the preventive potential for dementia has not declined over the last two decades.

## Background

In recent years, it has become clear that cardiovascular factors and low education are risk factors of dementia [1–3]. As advocated by the recent World Alzheimer report [3], most of these factors are potentially modifiable, which provides an opportunity for prevention of dementia. The magnitude of this potential for prevention can be estimated via the population attributable risk (PAR) [4].

Few studies have estimated PAR for modifiable risk factors of dementia [5–10]. However, results have been inconsistent due to methodological variation. For instance, some studies estimated PAR using data from multiple different sources, which precludes proper adjustment for risk factors and only yields indirect approximations of PAR [6, 8, 10]. Nevertheless, the combined PAR of potentially modifiable risk factors of dementia has been estimated to range from 8.4–50.7 % [6–9].

At the same time, converging evidence, including from our own study, suggests that the incidence of dementia has declined over the last decades [11–15]. Presumably, this decline has been triggered by a better control of cardiovascular risk and improved education [11–15]. If this is indeed the case, it is conceivable that previously published PARs of modifiable risk factors for dementia have now become an overestimation. There is thus an urgent need for contemporary data on PAR of modifiable risk factors of dementia, because such updated knowledge can better inform public health priorities on preventive strategies against dementia. In this study, we provide direct and contemporary estimates of PAR of modifiable risk factors of dementia. Additionally, we investigate how this PAR has changed across the last two decades.

## Methods

### Setting and study population

This study was embedded within the Rotterdam Study, a prospective population-based cohort study. In 1990, all residents aged 55 and older residing in Ommoord, a district of Rotterdam, the Netherlands, were invited. Of the 10,215 invited inhabitants, 7,983 agreed to participate in the baseline examinations. In 2000, the cohort was extended: again all residents aged 55 and older of the same district were invited, except for the participants that were already participating in the original cohort. Of the 4,472 invitees, 3,011 agreed to participate. Follow-up examinations take place every 3 to 4 years [16].

From the original cohort, we excluded 455 participants because they were not properly screened for dementia, 482 participants because they had prevalent dementia, and 43 participants for lack of follow-up information on the dementia diagnosis. Finally, 7,003 participants were included in the analyses. In the extended cohort, we excluded 29 participants because they had prevalent dementia and 29 for lack of follow-up information on the dementia diagnosis. Finally, 2,953 participants were included in the analysis. Even though a part of the original cohort still participated in 2000, those participants were only included in the original, and not in the extended cohort. We aimed to avoid overlapping individuals, since such a correlation would limit the assessment of cohort effects.

The Rotterdam Study has been approved by the medical ethics committee according to the Population Study Act Rotterdam Study and written informed consent was obtained.

### Selection and measurement of risk factors

Potentially causal and modifiable risk factors for dementia were chosen on the basis of previous literature [1–3]. The following risk factors were selected: overweight and obesity, hypertension, diabetes mellitus, unfavorable cholesterol levels, smoking, and low educational level.

All risk factors were measured at baseline. Weight and height were measured at the research center visit and body mass index (BMI) was calculated as weight in kilograms divided by length in meters squared. BMI was categorized into four categories: underweight (<18.5), normal weight (18.5–25), overweight (25–30), and obesity (>30). Blood pressure was measured in sitting position on the right arm and calculated as the average of two measurements using a random-zero sphygmomanometer. Hypertension was defined as a blood pressure ≥140/90 mmHg or use of blood pressure lowering medication, prescribed for the indication of hypertension. Diabetes mellitus was defined as a fasting serum glucose level ≥7.0 mmol/L, non-fasting serum glucose level ≥11.1 mmol/L, or use of anti-diabetic medication. Serum glucose, total cholesterol, and high-density lipoprotein (HDL)-cholesterol levels were acquired by an automated enzymatic procedure (Boehringer Mannheim System). Medication use, educational level, and smoking habits were assessed by interview. For people not taking lipid-lowering medication, total cholesterol/HDL-cholesterol ratio was divided in quartiles using the lowest category as the reference category. People using lipid-lowering medication were added as the fifth category and also compared to the reference category. Educational level was categorized as low (primary education or lower vocational education), intermediate (secondary education or intermediate vocational education), and high educational level (higher vocational education or university). Smoking habits were categorized as current, former, and never smoking.

Although stroke, coronary heart disease, heart failure, and atrial fibrillation are cardiovascular diseases that may result from cardiovascular risk factors and can be modified only via secondary prevention, several studies have shown that these diseases are related to dementia or cognitive impairment, independently of cardiovascular risk factors [17–20]. We therefore assessed the PAR of these factors in an additional analysis. A history of stroke, coronary heart disease (myocardial infarction or revascularization procedure), heart failure, and atrial fibrillation was evaluated using home interviews and confirmed by reviewing medical records [21, 22].

### Assessment of dementia

Participants were screened for dementia at baseline and at follow-up examinations using a three-step protocol [11]. Screening was done using the Mini-Mental State Examination (MMSE) and the Geriatric Mental Schedule (GMS) organic level. Screen-positives (MMSE <26 or GMS organic level >0) subsequently underwent an examination and informant interview with the Cambridge Examination for Mental Disorders in the Elderly [11]. Participants who were suspected of having dementia underwent extra neuropsychological testing if necessary. Additionally, for persons not visiting the research center, the total cohort was continuously monitored for dementia through computerized linkage of the study database and digitized medical records from general practitioners and the Regional Institute for Outpatient Mental Health Care. When information on neuroimaging was required and available, it was used for decision making on the diagnosis. Ultimately, a consensus panel, led by a neurologist, decided on the final diagnosis in accordance with standard criteria for dementia (DSM-III-R) [11]. Both the original and the extended cohort were followed for dementia until 10 years after baseline examinations: until 2000–2003 for the original cohort and until 2010–2012 for the extended cohort. Follow-up for dementia was complete for 99.5 % of potential person-years in the original cohort and for 98.5 % of potential person-years in the extended cohort.

### Statistical analyses

We imputed missing data on the investigated risk factors (3.3 % in the original cohort and 6.7 % in the extended cohort) using the mean of five imputations. Missing data were imputed on age, sex, and all other investigated risk factors. Differences between the original and the extended cohort were calculated using logistic regression models, adjusting for age and sex where appropriate. Analyses were performed using statistical software package SPSS 20.0.

We calculated PARs and corresponding 95 % confidence intervals (CIs) using the Interactive Risk Attributable Program (US National Cancer Institute) [23]. This metric is also referred to as population attributable fraction, or PAR%. We provide logit transformed 95 % CIs accompanying the PARs as these are better interpretable and more stringent with regard to our combined PAR estimates [24]. The PAR was estimated and adjusted for confounding factors according to the following formulae:

1 $$ \mathrm{P}\mathrm{A}\mathrm{R}=1-{\displaystyle {\sum}_{i=1}^{\mathrm{I}\ }}{\displaystyle {\sum}_{j=1}^J}{\rho}_{ij}{R}_{i\Big|j}^{-1} $$

where

2 $$ \left.Ri\right|j=\frac{ \Pr \left(D=1\Big|X= xi,C=cj\right)}{ \Pr \left(D=1\Big|X=x1,C=cj\right)} $$

and

3 $$ {\uprho}_{\mathrm{i}\mathrm{j}} = \Pr \left(\mathrm{X}={\mathrm{x}}_{\mathrm{i}},\mathrm{C}={\mathrm{c}}_{\mathrm{j}}\left|\mathrm{D}=1\right.\right) $$

with *D* = 1 denoting presence of disease, *X* denoting exposure with *i* levels, and *C* denoting a confounder with *j* levels. Participants were censored at date of dementia, date of death, or last date of follow-up, whichever came first. The relative risk was estimated from a multivariable Poisson model [23]. First, we calculated the PAR for overweight and obesity, hypertension, diabetes mellitus, unfavorable cholesterol levels, smoking, and low educational level. Second, we calculated the PAR of stroke, coronary heart disease, heart failure, and atrial fibrillation. We calculated the PAR for each risk factor separately as well as the combined PAR per cohort. Many risk factors have a coinciding effect on the etiology of dementia. Therefore, the combined PAR cannot be estimated by the sum of the separate PARs as this would lead to an overestimation. The advantage of the statistical program used in this study is that it takes into account that a disease case can simultaneously be attributed to more than one risk factor. Hence, it takes into account the overlap between the PARs of each risk factor when estimating the combined PAR. The combined PAR included the PARs of modifiable risk factors which were associated to dementia in the expected direction in our study, meaning the relative risk was above one. PARs cannot be calculated using a relative risk below one as this will result in a PAR which cannot be interpreted [25].

Every PAR was adjusted for age, sex, and all other risk factors included in the model. Age was added per 5-year categories into the models. Because of a small number of people in the oldest age categories, the oldest age category of both cohorts comprised people 85 years of age and older.

## Results

### Baseline characteristics

Baseline characteristics of the two study populations are provided in Table 1. During a mean follow-up of 8.3 years (standard deviation (SD) 2.9), 624 participants of the original cohort developed dementia and 1,756 participants died. During a mean follow-up of 8.9 years (SD 2.3), 145 participants of the extended cohort developed dementia and 429 participants died. Cut-offs for quartiles of total cholesterol/HDL cholesterol ratio were: 4.1, 5.0, and 6.1 in the original cohort and 3.6, 4.3, and 5.2 in the extended cohort. Participants in the extended cohort were younger and had higher educational levels than those in the original cohort. Overweight and obesity, hypertension, diabetes mellitus, use of lipid-lowering medication, former smoking, and stroke were more prevalent in the extended cohort. Additionally, people had lower total cholesterol levels, higher HDL-cholesterol levels, and less heart failure and coronary heart disease in the extended cohort than in the original cohort (Table 1).

**Table 1:** Baseline characteristics of the original and the extended cohort of the Rotterdam Study

	RS-I	RS-II	*P* value for difference
	N = 7,003	N = 2,953	
Age, years	69.4 (9.1)	65.0 (8.3)	<0.001
Sex, female	4187 (59.8 %)	1661 (56.2 %)	0.15
Body mass index			
<18.5	69 (1.0 %)	15 (0.6 %)	0.65
18.5–25	2486 (37.5 %)	790 (29.9 %)	Reference
25–30	3097 (46.7 %)	1286 (48.6 %)	<0.001
>30	977 (14.7 %)	555 (21.0 %)	<0.001
Hypertension	3793 (56.3 %)	1688 (61.9 %)	<0.001
Diabetes mellitus	727 (10.5 %)	319 (10.9 %)	0.001
Total cholesterol, mmol/L	6.6 (1.2)	5.8 (1.0)	<0.001
HDL cholesterol, mmol/L	1.3 (0.4)	1.4 (0.4)	0.004
Lipid lowering medication	164 (2.3 %)	367 (12.5 %)	<0.001
Smoking			
Former	2848 (41.7 %)	1381 (47.4 %)	0.001
Current	1560 (22.8 %)	678 (23.3 %)	0.60
Educational level			
Intermediate	2540 (37.3 %)	1429 (49.4 %)	<0.001
Low	3700 (54.3 %)	975 (33.7 %)	<0.001
Stroke	175 (2.5 %)	94 (3.2 %)	<0.001
Coronary heart disease	535 (8.4 %)	163 (6.1 %)	0.04
Heart failure	220 (3.3 %)	31 (1.2 %)	0.01
Atrial fibrillation	316 (4.9 %)	79 (3.6 %)	0.44

Data are presented as means (standard deviations) or numbers (percentages). Percentages are calculated without missing data. Abbreviations: RS-I, Rotterdam Study I, original cohort; RS-II, Rotterdam Study II, extended cohort; N , number of participants; HDL, High density lipoprotein. Differences between the original and extended cohort were calculated using logistic regression models, adjusting for age and sex where appropriate

### Population attributable risk (PAR)

In the original cohort, smoking (PAR, 0.07; 95 % CI, 0.02–0.23) and lower educational level (PAR, 0.07; 95 % CI, 0.00–0.90) had the largest PARs, followed by diabetes mellitus (PAR, 0.04; 95 % CI, 0.01–0.09), hypertension (PAR, 0.04; 95 % CI, 0.00–0.44) and higher total cholesterol/HDL cholesterol ratio (PAR, 0.03; 95 % CI, 0.00–0.73). Overweight and obesity were not related to dementia in the expected direction. The combined PAR in the original cohort was 0.23 (95 % CI, 0.05–0.62; Table 2).

**Table 2:** Population attributable risk (PAR) of risk factors in the original and extended cohort

	RS-I			RS-II		
	n/N 624/7,003			n/N 145/2,953		
	HR (95 % CI)	PAR (95 % CI) per category	Combined PAR per risk factor (95 % CI)	HR (95 % CI)	PAR (95 % CI) per category	Combined PAR per risk factor (95 % CI)
Body mass index			NA			NA
<18.5	1.25 (0.62–2.56)	0.00 (0.00–0.08)		1.41 (0.19–10.49)	0.00 (0.00–0.67)	
25–30	0.96 (0.81–1.15)	NA		0.90 (0.61–1.32)	NA	
>30	0.84 (0.65–1.08)	NA		0.81 (0.48–1.36)	NA	
Hypertension	1.07 (0.89–1.27)		0.04 (0.00–0.44)	1.25 (0.81–1.94)		0.16 (0.02–0.62)
Diabetes mellitus	1.31 (1.05–1.63)		0.04 (0.01–0.09)	1.51 (0.96–2.37)		0.06 (0.02–0.19)
Cholesterol/HDL cholesterol			0.03 (0.00–0.73)			NA
Quartile 2	1.04 (0.83–1.30)	0.01 (0.00–0.82)		0.64 (0.39–1.04)	NA	
Quartile 3	1.08 (0.86–1.35)	0.02 (0.00–0.30)		0.86 (0.53–1.39)	NA	
Quartile 4	1.04 (0.83–1.32)	0.01 (0.00–0.70)		0.72 (0.42–1.21)	NA	
Lipid lowering medication	0.64 (0.28–1.45)	NA		1.03 (0.60–1.74)	0.00 (0.00–1.00)	
Smoking			0.07 (0.02–0.23)			NA
Former	1.10 (0.90–1.34)	0.03 (0.00–0.23)		1.00 (0.68–1.48)	0.01 (0.00–1.00)	
Current	1.33 (1.04–1.71)	0.04 (0.02–0.10)		0.86 (0.48–1.52)	NA	
Educational level			0.07 (0.00–0.90)			0.12 (0.00–0.89)
Intermediate	0.99 (0.68–1.45)	NA		1.01 (0.57–1.78)	0.00 (0.00–1.00)	
Low	1.13 (0.78–1.63)	0.08 (0.00–0.66)		1.32 (0.73–2.36)	0.11 (0.02–0.51)	
Total			0.23 (0.05–0.62)			0.30 (0.06–0.76)

Estimates represent hazard ratios (95 % CIs) and population attributable risks (PARs; 95 % CIs). Models were adjusted for age, sex, and for body mass index, hypertension, diabetes mellitus, total cholesterol/HDL cholesterol ratio, lipid-lowering medication, smoking, and educational level, if appropriate. For some risk factors, PARs could not be calculated as these risk factors were not related to dementia in the expected direction; these PARs are therefore not applicable (indicated with NA) [25]

*Abbreviations: RS-I* Rotterdam Study I, original cohort, *RS-II* Rotterdam Study II, extended cohort, *HR* Hazard ratio, *CI* Confidence interval, *n* Number of cases, *N* Number of people at risk, *PAR* Population attributable risk, *NA* Not applicable, *HDL* High density lipoprotein

In the extended cohort, hypertension (PAR, 0.16; 95 % CI, 0.02–0.62), lower educational level (PAR, 0.12; 95 % CI, 0.00–0.89), and diabetes mellitus (PAR, 0.06; 95 % CI, 0.02–0.19) had the largest PARs. Overweight and obesity, higher total cholesterol/HDL-cholesterol ratio, and smoking were not related to dementia in the expected direction. The combined PAR in the extended cohort was 0.30 (95 % CI, 0.06–0.76; Table 2).

When cardiovascular diseases were included into the model, the combined PAR was 0.25 (95 % CI, 0.07–0.62) in the original cohort and 0.33 (95 % CI, 0.07–0.77) in the extended cohort. Atrial fibrillation and stroke had the largest PARs in the original cohort, whereas coronary heart disease and stroke had the largest PARs in the extended cohort (Table 3).

**Table 3:** Population attributable risk (PAR) of cardiovascular diseases in the original and extended cohort

	RS-I		RS-II	
	n/N 624/7,003		n/N 145/2,953	
	HR (95 % CI)	Combined PAR per risk factor (95 % CI)	HR (95 % CI)	Combined PAR per risk factor (95 % CI)
Total without CVD		0.23 (0.05–0.62)		0.30 (0.06–0.76)
Stroke	1.43 (1.00–2.04)	0.02 (0.00–0.05)	1.70 (0.86–3.37)	0.03 (0.01–0.13)
Coronary heart disease	1.00 (0.73–1.38)	0.00 (0.00–1.00)	1.38 (0.79–2.43)	0.03 (0.00–0.20)
Heart failure	0.87 (0.59–1.28)	NA	0.33 (0.04–2.39)	NA
Atrial fibrillation	1.32 (0.99–1.77)	0.02 (0.01–0.06)	0.36 (0.13–0.97)	NA
Total including CVD		0.25 (0.07–0.62)		0.33 (0.07–0.77)

Estimates represent hazard ratios (95 % CIs) and population attributable risks (PARs; 95 % CIs). Models were adjusted for age and sex, and for body mass index, hypertension, diabetes mellitus, total cholesterol/HDL cholesterol ratio, lipid-lowering medication, smoking, educational level, stroke, coronary heart disease, heart failure, and atrial fibrillation, if appropriate. For some risk factors, PARs could not be calculated as these risk factors were not related to dementia in the expected direction; these PARs are therefore not applicable (indicated with NA) [25]

*Abbreviations: RS-I* Rotterdam Study I, original cohort, *RS-II* Rotterdam Study II, extended cohort, *n* Number of cases, *N* Number of people at risk, *HR* Hazard ratio, *CI* Confidence interval, *PAR* Population attributable risk; CVD, Cardiovascular disease; NA, Not applicable

## Discussion

Within the population-based Rotterdam Study, we found that about one quarter to one third of dementia cases could potentially be prevented through optimal prevention or treatment of cardiovascular risk factors and diseases and improvement of educational level. This proportion has not declined between the original cohort of 1990–2000 and the extended cohort of 2000–2010, although we did observe a shift in the relative importance of individual factors.

Before these results can be interpreted, some methodological issues need to be mentioned. The strengths of the present study are the prospective, population-based design and almost complete dementia case finding. Additionally, information on a relatively wide range of risk factors was assessed, allowing the calculation of PARs on various potentially modifiable risk factors. Furthermore, a statistical approach, which takes into account the interaction between risk factors when estimating the combined PAR, was used.

We recognize that our study also has limitations. PAR calculations should always be interpreted with caution as they rely on assumptions which may be improbable in practice [26, 27]. For instance, the model assumes that a risk factor can be successfully treated in every person and that removing one risk factor would not induce changes in the other factors, which is unlikely. Furthermore, PAR calculations assume that, when a risk factor is successfully treated, the harming effect of the risk factor completely disappears, which too is unlikely as risk factors might have already caused irreversible damage before treatment is started. Moreover, if we prevent a dementia case caused by these cardiovascular risk factors, this person might still suffer from dementia caused by other factors. Therefore, the proportion that can actually be prevented in practice might be lower than suggested by the PAR [26, 27]. We selected potentially causal and modifiable risk factors of dementia based on previous literature [1–3]. However, it should be noticed that, within this study, this causal relationship could not be established. In particular, findings related to cardiovascular diseases should be interpreted with caution as these may have an association with dementia through shared risk factors. Paradoxically, treatment might even increase the occurrence of dementia, if the people that are treated live longer.

In our study, we did not find many statistically significant associations between these risk factors and the risk of dementia. This might have various explanations. First, dementia is a multifactorial disease and the effect of a single risk factor can therefore be relatively small and non-significant. However, the preventive potential of all risk factors combined can still be substantial. Second, the sample size was limited, which might have prevented the finding of statistically significant results because of low power and because smaller subgroups for the risk factors, such as untreated and treated hypertension, could not be formed. Third, baseline risk factors were used, whereas the risk factor profile might have changed during follow-up. This may have weakened the associations. Fourth, the effect of cardiovascular risk factors might be small because of the competing risk of cardiovascular mortality. People might die due to those risk factors before dementia occurs. Fifth, it has been suggested that the effect of several cardiovascular risk factors on the risk of dementia changes with increasing age. Studies have shown that obesity, hypertension, and high cholesterol levels were only related to an increased risk of dementia when assessed at midlife [28–30]. Since the participants in our study populations were older, we were not able to assess such midlife effects, possibly leading to an underestimation of our results. Although our data collection was extensive, we did not have information on some important modifiable risk factors, such as physical activity, dietary habits, depression, and social engagement, which might have led to an underestimation of our results. We imputed missing data on investigated risk factors, which may have introduced some misclassification. However, we hypothesized that excluding participants with missing data from our analyses would have resulted in more bias, given that missing data usually does not occur at random. A final limitation is that most participants of the Rotterdam Study are Caucasians and live in a middle-income district of Rotterdam, which limits the generalizability of our results.

We found that the PAR of modifiable risk factors of dementia was about one quarter to one third and has not declined across the last two decades. These findings have several implications. First, from a public health point of view, this suggests that despite the seemingly declining incidence of dementia, the potential for further reduction of dementia is still substantial. Second, it remains pivotal to find novel risk factors that can explain the remaining two thirds of dementia cases. Third, even though the combined PAR did not decline, we did find that the contribution of individual risk factors had changed across the two decades. For example, the PARs of hypertension and to a lesser extent of diabetes were higher in the extended cohort. Although treatment and preventive options for cardiovascular risk factors have improved over the past decades, the prevalence of various cardiovascular risk factors, such as hypertension, diabetes, and obesity has increased [31–33]. Correspondingly, we found that participants in the extended cohort had a higher prevalence of these risk factors, partly explaining the higher PARs. Conversely, we found a decline in the PARs of other cardiovascular risk factors, such as smoking and unfavorable cholesterol levels. Smoking had a large effect on the burden of dementia in the original cohort, which decreased dramatically in the extended cohort. Successful anti-smoking campaigns are an obvious explanation for these findings. A counterargument, however, is that the prevalence of current smokers was comparable between the two cohorts. The reduction of the effect of unfavorable cholesterol levels might be explained by the large increase in use of lipid-lowering medication in the extended cohort. Another interesting observation was the effect of educational level, which was higher in the extended cohort than in the original cohort. Conventionally, educational attainment is considered a reflection of cognitive reserve built up earlier in life. However, this has not always been the case in older Dutch generations, where many people were unable to achieve their educational potential due to the Second World War. This might have led to a discrepancy between educational attainment and corresponding cognitive reserve. In the original cohort of the Rotterdam Study, this phenomenon might have been more pronounced, explaining the higher prevalence but lower PAR of low educational level compared to the extended cohort.

As for cardiovascular diseases, atrial fibrillation had an effect on the burden of dementia in the original cohort, but not in the extended cohort. These results might be explained by improved prevention of ischemic stroke in patients with atrial fibrillation [34]. In contrast, we found a stronger effect of stroke and coronary heart disease in the extended cohort than in the original cohort. This might seem counter-intuitive, since preventive and treatment options for these diseases have improved in the same time-period. However, these findings could be explained by the fact that because of improved treatments, people with cardiovascular disease live longer and therefore are at an increased risk of developing dementia [35].

The combined PAR we found is very much in line with a recent report that was based on meta-analyses of studies, which mostly used data from the nineties [8]. We calculated PARs directly using original data from 1990–2000 and 2000–2010 and, more importantly, took into account interaction between risk factors. Our study therefore adds important veracity to the estimates of PAR. However, given that PAR calculations rely on theoretical assumptions, future research is necessary to observe the actual effect of risk factor improvement on the risk of dementia. Furthermore, other studies should also focus on identifying novel modifiable risk factors for dementia.

## Conclusions

We found that the potential of prevention of dementia through proper control of modifiable risk factors is about one quarter to one third and has not declined over the last two decades. As this is currently one of the main options to diminish the burden of dementia, public health interventions are urgently needed.

## Abbreviations

BMI: Body mass index
CI: Confidence interval
HDL: High-density lipoprotein
PAR: Population attributable risk
SD: Standard deviation
